# Supplementary material for: Evidence for a three-dimensional quantum spin liquid in PbCuTe2O6
Source: Nat Commun. 2020 May 11;11:2348. doi: 10.1038/s41467-020-15594-1 (PMC7214440; doi:10.1038/s41467-020-15594-1)
Supplement: Supplementary file 1 — Supplementary Information [file 41467_2020_15594_MOESM1_ESM.pdf]

Evidence for a three-dimensional quantum spin liquid in $PbCuTe_2O_6$

Chillal et al.

February 18, 2020

Supplementary Note 1: DC susceptibility

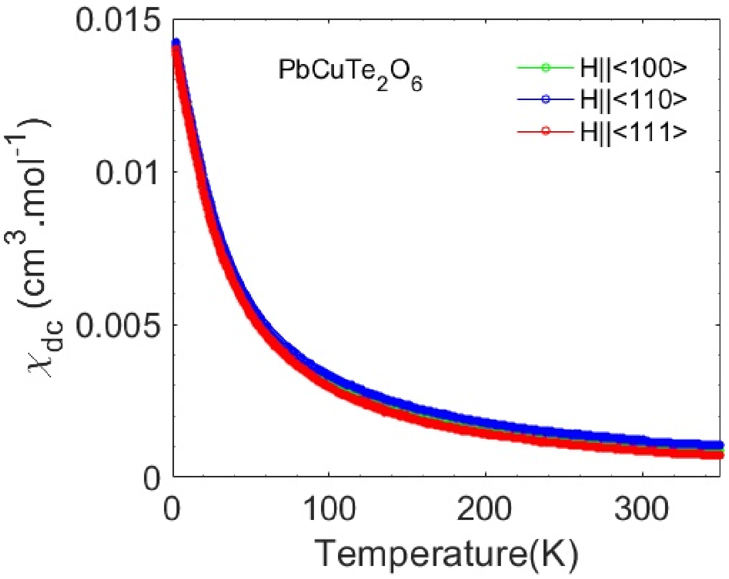

Supplementary Figure 1: The static magnetic susceptibility measured on a single crystal of $PbCuTe_2O_6$. With magnetic field applied parallel to the three inequivalent directions of the cubic system, $[1,0,0]$, $[1,1,0]$ and $[1,1,1]$. The sample is cooled in zero field and the susceptibility is measured as a function of temperature in a constant field of $H = 1$ T. The plot reveals that the susceptibility is nearly the same for all the directions indicating that the system can be considered magnetically isotropic.

In order to verify the isotropic nature of the Cu^{2+} moments in $PbCuTe_2O_6$, static (DC) susceptibility measurements on single crystal samples have been compared for three inequivalent magnetic field directions within the cubic system. For this purpose, the zero-field cooled moment was measured under a $H = 1$ T field applied parallel to the $[1,0,0]$, $[1,1,0]$ and $[1,1,1]$ crystallographic directions for temperatures in the range $T = 2$ K to 350 K. Supplementary Figure1 shows that the susceptibility displays no indication of any phase transitions down to 2 K. Furthermore the susceptibility is almost completely independent of the field direction. Therefore, we can conclude that the Cu^{2+} spin-$\frac{1}{2}$ moments in $PbCuTe_2O_6$ are isotropic in nature.

Supplementary Note 2: Muon spin relaxation spectroscopy

μSR is an invaluable technique to probe the magnetic ground state as it is extremely sensitive to the local magnetic environment. Supplementary Figure 2 shows the zero field muon depolarization spectra of $PbCuTe_2O_6$ from 5 K down to 0.02 K. A classic evidence of long-range order is revealed when spontaneous oscillations appear in the muon decay spectrum due to the precession of muon around an internal

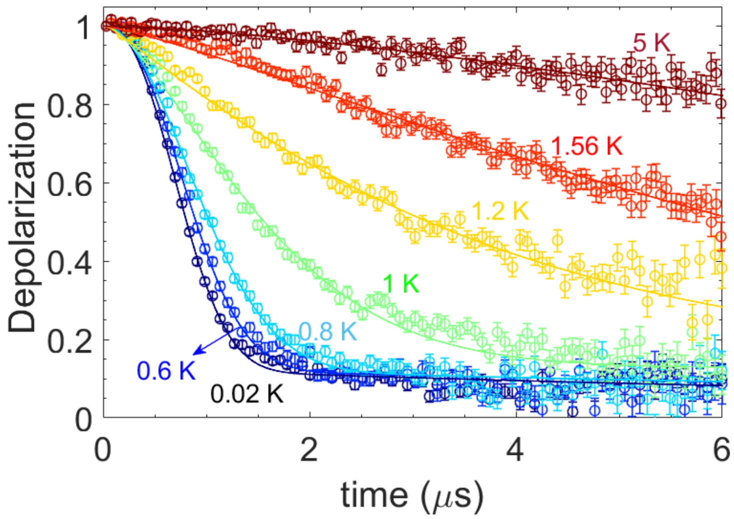

Supplementary Figure 2: The zero-field μSR spectroscopy of $PbCuTe_2O_6$. The μSR depolarization spectra as a function of temperature measured in zero field along with statistical error bars. At all the temperatures, the spectra decays to reach the background level at longer time scales. At base temperature, this is clearly observed already for timescales above $2\ \mu$S pointing to the purely dynamic nature of the ground state in $PbCuTe_2O_6$.

Atom	x/a	y/b	z/c
Te	0.3379(7)	0.9213(5)	0.0570(5)
Pb1	0.0576(1)	0.0576(1)	0.0576(1)
Pb2	0.875	0.875	0.875
Cu	0.125	0.7742(6)	0.0242(6)
O1	0.5826(7)	0.9261(1)	0.3718(4)
O2	0.2668(1)	0.8128(2)	0.9775(8)
O3	0.2233(7)	0.9817(3)	0.1276(6)

Supplementary Table 1: The atomic coordinates of polycrystalline $PbCuTe_2O_6$ from synchrotron diffraction. Measured at 300 K, these parameters are obtained by refining the powder diffraction pattern by a cubic structure with $P\,4_1 32$ space group and lattice parameter $a = 12.4968(4)$ Å.

magnetic field resulting from the magnetic long-range order at the time of decay. In powder samples, static magnetism also leaves nonzero polarization at long time scales due to spherical symmetry of the internal fields so that $\frac{1}{3}^{rd}$ of the fields are directed along the initial muon polarization. However, neither of these features are visible in case of $PbCuTe_2O_6$ indicating the absence of long-range magnetic order or spin glass freezing. Nevertheless there is evidence for enhanced magnetic correlations as the relaxation rate of the muon spin polarization, directly related to the spin dynamics, slows down rapidly as the sample is cooled below 1K and becomes constant below 0.6 K. This suggests persistent spin dynamics in the ground state of $PbCuTe_2O_6$. Thus, the combination of neutron diffraction and μSR confirm the absence of static magnetic order in $PbCuTe_2O_6$ down to 0.02 K and the purely dynamic nature of the ground state is established.

Supplementary Note 3: Single crystal growth and structure refinement

The starting material for crystal growth was prepared from high purity powder of PbO (99.99%, Alfa Aesar), CuO (99.995%, Alfa Aesar) and TeO_2 (99.9995%, Alfa Aesar) mixed thoroughly in the 1:1:2 molar ratio. After mixing, powder of stoichiometric composition was calcined in a platinum crucible under flowing argon atmosphere in a vacuum furnace for 12 hours at 510°C, twice with intermediate grinding. The resultant polycrystalline sample was characterized for phase purity using both x-ray and neutron powder diffraction. Supplementary Figure3a shows the synchrotron diffraction of the powder

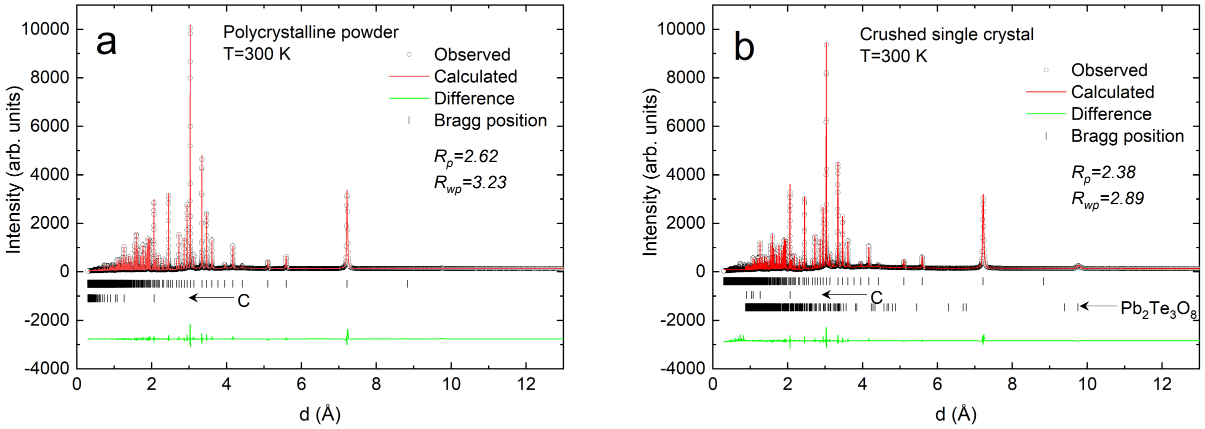

Supplementary Figure 3: **The diffraction patterns of polycrystalline and crushed single crystals of $PbCuTe_2O_6$ with synchrotron radiation.** Measurements are performed on samples diluted with 3 parts of diamond powder (C) at room temperature with photons of wavelenth 0.496 Å. **a** The polycrystalline diffraction pattern refined by considering the cubic structure based on $P\,4_1 32$ space group and lattice parameter $a = 12.4968(4)$ Å. **b** The diffractin pattern from crushed single crystals refined with the same structure as the polycrystalline samples where both the diamond powder and non-magnetic impurity are refined in profile-matching mode.

sample measured with a photons of wavelength 0.496 Å. The pattern is refined with nuclear structure of $P\,4_1 32$ space group and lattice constant $a = 12.4968(4)$ Åand Supplementary Table 1 lists the atomic coordinates.

The powder was then pulverized and packed into a cylindrical rubber tube and pressed hydrostatically up to 2000 bars in a cold-isostatic-pressure (CIP) machine. The pressed rod was then hung in the vacuum furnace and sintered in flowing argon for 12 hours at 550°C to form a dense cylindrical rod with diameter about 0.6 cm and length $7 - 8$ cm. Crystal growth was carried out in an optical image furnace (Crystal Systems Corp., FZ-T 10000-H-VI-VPO) equipped with four 150 W Tungsten halide lamps focused by ellipsoidal mirrors. Crystal growths were performed in under 0.1 MPa Argon atmosphere at a growth rate of 1.0 mm per hour. Single crystallinity of the as-grown crystal was checked by X-ray Laue and polarized optical microscopy. A piece of the crystal was ground and checked with x-ray powder diffraction (Brucker D8) for phase purity. We have grown several batches of $PbCuTe_2O_6$ single crystals, all of which consistently reveal $5 - 10\%$ of a non-magnetic impurity $Pb_2Te_3O_8$. Supplementary Figure. 3b shows the diffraction pattern on a finely ground single crystal sample measured with synchrotron radiation similar to the ceramic powder where, the refinement (lattice parameter $a = 12.4967(8)$ Åfor $PbCuTe_2O_6$) includes the impurity phase $Pb_2Cu_3O_8$ in profile-matching mode. Although this impurity is also present in the as-grown polycrystalline sample, it is always less than 5%.

Supplementary Note 4: Low temperature characterization of single crystal

Heat capacity, μSR studies in Ref. [1, 2] and our neutron diffraction (Figure.2 of the main text) investigation on the powder samples confirm the absence of magnetic phase transitions in $PbCuTe_2O_6$ down to 0.02 K. In this work, we have carried out similar investigation on the single crystals including heat capacity, thermal expansion and thermal conductivity to probe the low temperature properties. Results of these studies will be discussed in more detail in another publication.

In addition, to confirm the absence of magnetic long-range order in the single crystal of $PbCuTe_2O_6$, we have performed neutron diffraction on the same single crystal that was used for measuring the inelastic scattering. The diffraction patterns were measured on the WISH diffractometer (white neutron beam) at temperatures $T < 0.1$ K and at 2 K for three orientations of the crystal while focusing the flux on two types of peaks where magnetic order could be expected: 1) equivalent positions $[0.3, 0.3, 1.69]$ where inelastic spectrum shows the intensity maxima (Fig. 3e & Fig. 4a-b of the main text), 2) $[0, 0, l]$ peaks at which the iso-structural compound $SrCuTe_2O_6$ shows magnetic Bragg peaks. Supplementary Figure4a, shows Laue images in the $[h, h, l]$ scattering plane at 0.1 K and 2 K in the vicinity where

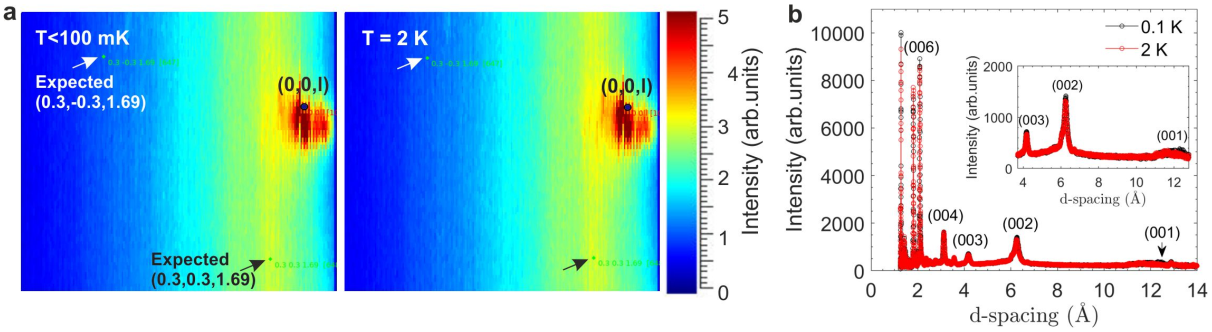

Supplementary Figure 4: Neutron diffraction patterns of single crystal of $PbCuTe_2O_6$ at $T < 0.1$ K and $T = 2$ K at WISH diffractometer. a The Laue pattern focusing on the peaks of type $[0.3, 0.3, 1.69]$ (positions of inelastic maxima) revealing no induced magnetic Bragg peak at low temperature at small wave vectors. **b** the intensities of peaks along $[0, 0, l]$ direction, showing the forbidden (001), (002) and (003) peaks (highlighted in the inset) due to multiple scattering at both the temperatures.

the $[0.3, 0.3, 1.69]$ reflection is expected. Here, the magnitude of the momentum transfer $|\mathbf{Q}|$ increases with respect to the distance from $[0, 0, l]$ and other nuclear peaks in this region are masked for clarity. The patterns at both temperatures are identical and no magnetic intensity is induced at $[0.3, 0.3, 1.69]$. Whereas, the $[0, 0, l]$ peaks (001), (002), (003) show an increment in the intensity at low temperature 0.1 K, as seen in the inset of Supplementary Fig. 4b. However, we should note that these peaks are forbidden due to the lattice symmetry ($P\,4_1 32$) and are also present at 2 K due to the large size of the single crystal resulting in multiple scattering. Nevertheless, these reflections are weak compared to the allowed Bragg reflections. Additionally, the increased intensities do not decay as the square of the momentum transfer (in which case, the highest magnetic peak should be $(0, 0, 1)$) and hence these peaks cannot be attributed to magnetic order in the system. Therefore, we can exclude the possibility of magnetic order in the single crystal of $PbCuTe_2O_6$, at least, at the most-likely wave vectors. However, we cannot rule out the possibility of structural changes involving only certain symmetries of the space group $P\,4_1 32$.

Supplementary Note 5: Low energy inelastic neutron scattering

Highly frustrated systems that undergo magnetic order at very low temperatures would result in their low-energy excitation spectra modulated to exhibit spin waves from the ordered moment. Therefore, the low energy part of the excitations in $PbCuTe_2O_6$ is investigated on both the powder and single crystal samples using the LET spectrometer. The Supplementary Fig. 5a&b show the spectra measured with incident energy of $E = 1.5$ meV and 1.3 meV for powder and single crystal respectively where the single crystal data is integrated over the $-2.35 \leqslant [0, 0, l] \leqslant -1$ r.l.u range . It is clear that both the spectra consistently exhibit only diffuse excitations down to 0.1 meV originating at $|\mathbf{Q}|$ ~0.8 Å^{-1} and have similar intensity modulations. Hence the absence of any magnons in $PbCuTe_2O_6$ is confirmed.

Supplementary Note 6: Comment on previous Density functional theory calculations in Ref. [2]

The energy mapping method applied in our density functional theory calculations employs fully charge self-consistent solutions for different spin configurations, therefore it includes at the density functional theory level the exchange contributions to arbitrary order. It is clear that this approach yields different and usually more reliable estimates than the second-order perturbation theory formula $J = \frac{4t^2}{U}$ used previously [2] which expresses only the antiferromagnetic superexchange contribution based on one virtual process. The latter approximation leaves out a large number of other exchange processes both from higher orders of perturbation theory as well as ferromagnetic contributions. The results from our DFT calculations yield interactions up to 4th nearest neighbour with a ratio of J_1:J_2:J_3:J_4 = 1.06:1:0.55:0.11 for the Curie-Weiss temperature of $\theta_{CW} = -23$ K. In contrast, the previous method finds only the first three neighbours to be relevant and the ratio of couplings is J_1:J_2:J_3 = 0.54:1:0.77. The difference

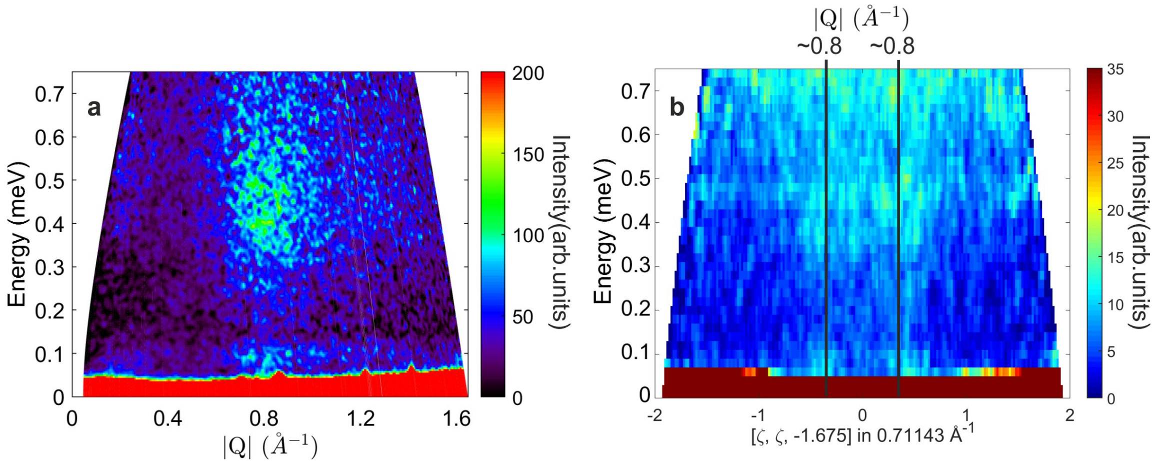

Supplementary Figure 5: **Excitation spectra of $PbCuTe_2O_6$ at low energy transfer in powder and single crystal at $T < 0.1$ K.** Measured at temperature $T < 0.1$ K on the LET spectrometer at the ISIS facility for, **a** powder sample plotted as a function of energy and momentum transfer measured with incident energy $E_i = 1.5$ meV and for, **b** single crystal plotted as a function of energy and wavevector along the $[h, h, 0]$-axis with scattering integrated over $-2.35 \leqslant [0, 0, l] \leqslant -1$ r.l.u in the perpendicular direction and $-0.1 \leqslant [h, -h, 0] \leqslant 0.1$ r.l.u in the vertical plane to include the peak maxima on the diffuse scattering sphere as seen in Fig. 4a of the main text.

between the two methods is evident in the spin susceptibility maps calculated using the PFFRG method based on the Hamiltonian from these two sets of couplings as shown in Supplementary Figure6. Although the static spin susceptibility based on the Hamiltonian from the perturbation theory method [2] also produces a ring at ~ 0.8 $\AA^{-1}$, as shown in Supplementary Figure6a, the maxima on the ring appear at $(1, 1, 1)$ and equivalent positions in the $[h, h, l]-$plane. This is clearly different from the experimental results where the diffuse ring is broad and the maxima are at positions such as $(0.3, 0.3, \pm 1.69)$ as nicely reproduced by the Hamiltonian from our DFT calculations (see Supplementary Fig. 6c). The discrepancy of the previous Hamiltonian is even clearer in the $[h, k, 0]-$plane (compare Supplementary Fig. 6b&d) where the double peak at $(\pm 1.69, \pm 0.3, 0)$ is missing. Supplementary Figure 6e&f show cuts through the data and the calculated spin susceptibility of the two Hamiltonians. The agreement between our Hamiltonian and the data is clearly much better than that of the previous Hamiltonian [2].

Supplementary Note 7: Ground state properties of classical Heisenberg models on the hyperkagome lattice

As discussed in the main text, the origin of the spin fluctuations in $PbCuTe_2O_6$ can be explained within a classical Heisenberg model that includes only the triangular J_1 and hyperkagome J_2 interactions even though this material also features additional J_3 and J_4 couplings. In particular, the streaks in the momentum resolved classical susceptibility of the J_1-J_2 model resemble the PFFRG quantum calculation of the full model, as shown for the $[h, h, l]-$plane in Figure 4e. The susceptibility in the $[h, k, 0]-$plane (Figure 4f) shows points of strong intensity since this plane cuts through the streaks.

This correlation profile can be compared with a classical Heisenberg model with J_2 hyperkagome interactions only, as it has previously been studied in Ref. [5]. The ground state of this model is macroscopically degenerate, i.e. for a system consisting of $L \times L \times L$ unit cells, the dimension of the degenerate manifold scales exponentially as $V = L^3$. As a consequence, the spin structure factor shows extended regions of strong intensity which fill a volume in momentum space, see Fig. 2 in Ref. [5]. Furthermore, in close similarity to classical pyrochlore systems, where an"ice rule" for each tetrahedron/triangle imposes a constraint on the local spin orientation, the structure factor exhibits pinch points.

When triangle J_1 interactions are added, further constraints are imposed on the possible ground states, which reduce the dimensionality of the degenerate ground state manifold. Particularly, it can be shown that the number of degenerate states becomes subextensive, i.e. it scales exponentially in the linear system size L [6]. As a consequence, the regions of strong intensity in the structure factor shrink

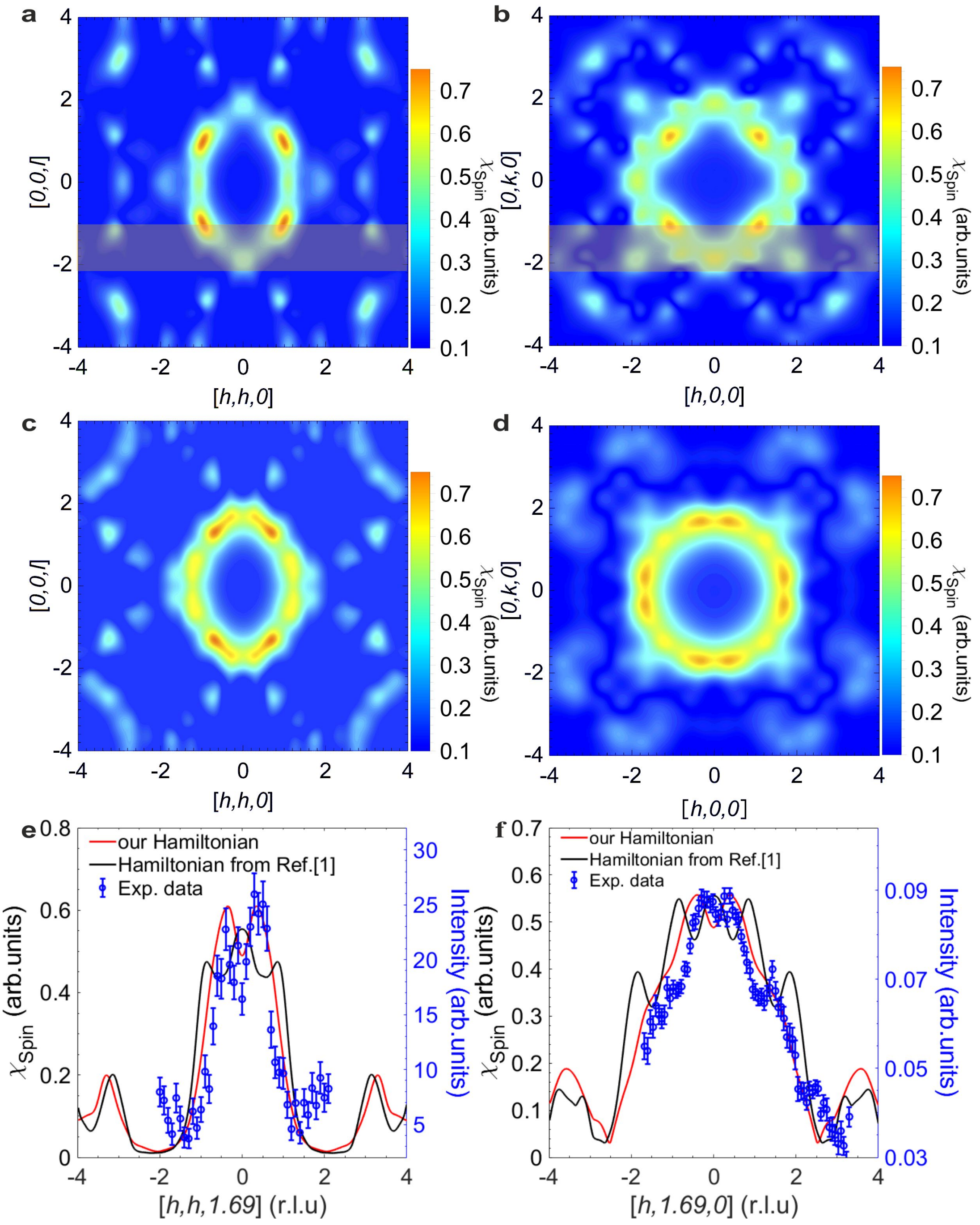

Supplementary Figure 6: **The static spin susceptibility profiles calculated using the PFFRG method.** From the Hamiltonian produced by: **a**-**b** second-order perturbation theory that expresses only the antiferromagnetic superexchange contribution based on one virtual process (Ref. [2]): and **c**-**d** the current work that employs the full potential local orbital basis set [3] and the generalized gradient approximation functional [4]. The former results produce a sharper diffuse ring in $[h, h, l]-$plane with maxima at $(1, 1, 1)$ and a diamond shaped profile in the $[h, k, 0]-$plane which do not agree with the experimental data. The latter results however show striking similarity with our neutron scattering maps. **e**-**f** Comparison between cuts through the experimental data and the expected magnetic intensity calculated for the two Hamiltonians using PFFRG. The data points were obtained by integrating the experimental data shown in Fig. 4**a**-**b** of the main text, over the respective shaded regions indicated in those figures. The same integration was performed over these calculations (see shaded region in **a**&**b**). The uncertainties represent statistical errors. As is clearly evident, our Hamiltonian gives much better agreement with the data than the previous Hamiltonian.

down to lines parallel to the $[1, 1, 1]$ (and symmetry related) directions. Interestingly, the susceptibility in Fig. 4e of main text still shows remainders of the pinch points such as the point $[0, 0, 2]$ where streaks of high intensity cross.

It is important to emphasize that even though the classical J_1-J_2 Heisenberg model only exhibits a subextensive ground state degeneracy, there is still a macroscopic number of low-energy states through which the system can fluctuate when quantum fluctuations are considered [6]. This still remains true when the weaker J_3 and J_4 interactions are added. While this further reduces the degeneracy to a finite number of states, the full manifold of the classical J_1-J_2 Heisenberg model still remains energetically nearby.

Supplementary References

[1] P. Khuntia, F. Bert, P. Mendels, B. Koteswararao, A. V. Mahajan, M. Baenitz, F. C. Chou, C. Baines, A. Amato, and Y. Furukawa. Spin Liquid State in the 3D Frustrated Antiferromagnet $PbCuTe_2O_6$: NMR and Muon Spin Relaxation Studies. *Phys. Rev. Lett.*, **116**, 107203, (2016).

[2] B. Koteswararao, R. Kumar, P. Khuntia, Sayantika Bhowal, S. K. Panda, M. R. Rahman, A. V. Mahajan, I. Dasgupta, M. Baenitz, K. H. Kim, and F. C. Chou. Magnetic properties and heat capacity of the three-dimensional frustrated $PbCuTe_2O_6$. *Phys. Rev. B*, **90**, 035141, (2014).

[3] K. Koepernik and H. Eschrig. Full-potential nonorthogonal local-orbital minimum-basis band-structure scheme. *Phys. Rev. B*, **59**, 1743, (1999).

[4] J. P. Perdew, K. Burke, and M. Ernzerhof. Generalized Gradient Approximation Made Simple. *Phys. Rev. Lett.*, **77**, 3865, (1996).

[5] J. M. Hopkinson, S. V. Isakov, H. -Y. Kee, and Y. B. Kim. Classical Antiferromagnet on a Hyperkagome Lattice. *Phys. Rev. Lett.*, **99**, 037201, (2007).

[6] Y. Iqbal et al. *in preparation.*
